# USO1 Coordinates Centriolar Satellites to Regulate Male Germ Cell Proliferation and Cell Cycle Progression

**DOI:** 10.3390/ijms26094274

**Published:** 2025-04-30

**Authors:** Xinyi Li, Peiyi Lin, Zaikuan Zhang, Runzhi Wang, Jing Cai, Xiaosong Feng, Zhihong Jiang, Shengming Xu, Yajun Xie

**Affiliations:** 1 The Ministry of Education Key Laboratory of Laboratory Medical Diagnostics, the College of Laboratory Medicine, Chongqing Medical University, Chongqing 400016, China; xinyili0505@163.com (X.L.); linpeiyihaha@163.com (P.L.); zhangzk@stu.cqmu.edu.cn (Z.Z.); leofeng1313@163.com (X.F.); 2024121634@stu.cqmu.edu.cn (Z.J.); 2024111610@stu.cqmu.edu.cn (S.X.); 2 National Talent Introduction Demonstration Base, the College of Basic Medicine, Harbin Medical University, Harbin 150081, China; yuchen3426@163.com (R.W.); 13765350516@163.com (J.C.)

**Keywords:** USO1, spermatogenesis, proliferation, centriolar satellites

## Abstract

The endoplasmic reticulum–Golgi apparatus system is an important organelle regulating male reproduction. USO1 vesicle transport factor (USO1), as an important molecule in this system, is a general vesicular transport factor and regulates various biological processes in vivo. However, the potential role of USO1 in mammalian testis development and spermatogenesis has not been investigated. We documented the presence of USO1 in mouse germ cells and its functional roles by generating *Uso1*-knockout germ cell lines. *Uso1* depletion suppressed cell proliferation and growth while stimulating apoptosis in GC1 and GC2 cells. In addition, the *Uso1* knockout blocked cell cycle progression and weakened DNA damage repair. Mechanistically, USO1 is associated with male reproduction by regulating the expression of genes involved in spermatogenesis, and we found evidence that USO1 is closely linked to centriolar satellites (CSs), which may play an important biological role. Overall, our findings reveal a vital role for USO1 in male fertility and offer a significant understanding of the functions of golgin proteins in reproductive biology.

## 1. Introduction

Mammalian spermatogenesis is subject to a series of complex and strict regulations. This process includes the proliferation and differentiation of spermatogonia, meiosis of spermatocytes, transformation of round spermatids into spermatozoa, and maturation of spermatozoa [1]. Spermatogonial stem cells maintain the stem cell pool through self-renewal and simultaneously generate progenitor spermatogonia for cell differentiation. Progenitor spermatogonia then differentiate into differentiating spermatogonia, followed by final mitosis to form preleptotene spermatocytes. After meiosis, two consecutive meiotic divisions occur. Homologous chromosome pairing, synapsis, homologous recombination, and chromatin remodeling occur during the first meiotic division. If these key processes are affected, it directly leads to cell cycle arrest and spermatogenesis failure [2,3,4,5]. After meiosis is complete, round haploid spermatids are formed, which become mature spermatozoa after undergoing drastic morphological and cytological changes.

USO1 was first identified in purified Golgi membranes [6]. Forming a myosin-like homodimer, USO1 has an N-terminal globular head, a smaller C-terminal containing four coiled-coil domains (CC1–CC4), and is terminated by a short acidic domain. As a member of the golgin family, the function of USO1 has been finely illustrated using in vitro systems. In addition to participating in membrane trafficking between the endoplasmic reticulum and Golgi apparatus [7,8,9,10,11], USO1 is also responsible for maintaining the structural integrity of the Golgi apparatus [12,13], regulating mitosis progression [14]. A recent study showed that in Drosophila testes, p115 is recognized as a downstream target of JAK/STAT signaling and is necessary for the maintenance of male germline stem cells (GSCs) [15]. Many endoplasmic reticulum and Golgi apparatus proteins cause male infertility by influencing the development of the sperm acrosome [16,17,18,19,20], but the role of USO1 in spermatogenesis and male reproduction remains largely unknown.

In this study, we demonstrate that the expression of USO1 is tissue- and cell-specific; it is highly expressed in mouse testes and germ cells. Silencing USO1 expression inhibits cell growth and induces cell cycle arrest while hindering spermatocyte DNA damage repair. The knockdown of *Uso1* alters the expression of reproduction-related genes in GC1 and GC2 cells. Furthermore, we made a new discovery: USO1 interacts with centriolar satellite proteins. Further studies are needed to clarify the mechanism by which USO1 regulates the centriolar satellite components. These studies would offer new insights into the biological functions of golgin proteins in mammalian gametogenesis.

## 2. Results

### 2.1. USO1 Expression in Mouse Germ Cells During Spermatogenesis

**Figure 1:**
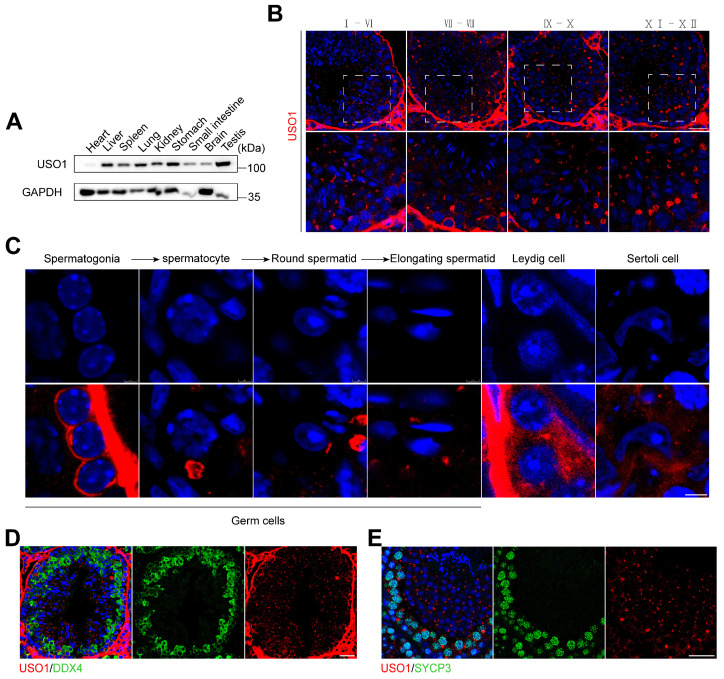
The expression of USO1 in male germ cells. (**A**) Western blotting was performed on tissue lysates of 8-week-old wild-type mice. Anti-USO1 and anti-GAPDH antibodies were used to analyze USO1 protein levels. (**B**,**C**) Immunostaining of USO1 (red) in testes sections prepared from wild-type mice at 8 weeks of age. The nuclei are stained with DAPI (blue). Scale bar = 50 μm. The lower panels show magnified images of single cells from the upper panels. Scale bar = 5 μm. (**D**,**E**) Immunostaining of USO1 (red) in testes sections showing DDX4-positive (green) germ cells and SYCP3-positive (green) spermatocytes. The nuclei are stained with DAPI (blue). Scale bar = 30 μm.

Initially, we examined the organ- and cell-specific expression pattern of USO1. The findings indicated that the USO1 protein was widely expressed throughout various tissues, yet it exhibited a particularly high abundance in the testes (Figure 1A). Next, we explored the localization of USO1 in mammalian spermatogenesis using immunostaining. We found that USO1 was localized in the cytoplasm of all germ cell types and had stronger signals in spermatogonia and spermatocytes (Figure 1B,C). To further validate the localization and expression of USO1 during spermatogenesis, immunohistochemical staining was performed using antibodies against the germ-cell-specific marker DDX4 and the synaptonemal complex component SYCP3 (Figure 1D,E). The co-localization patterns of USO1 with DDX4 and SYCP3 verified the spatiotemporal expression of USO1 in spermatogenic cells.

### 2.2. Uso1 Depletion Inhibits Germ Cell Proliferation and Impairs DNA Damage Repair

**Figure 2:**
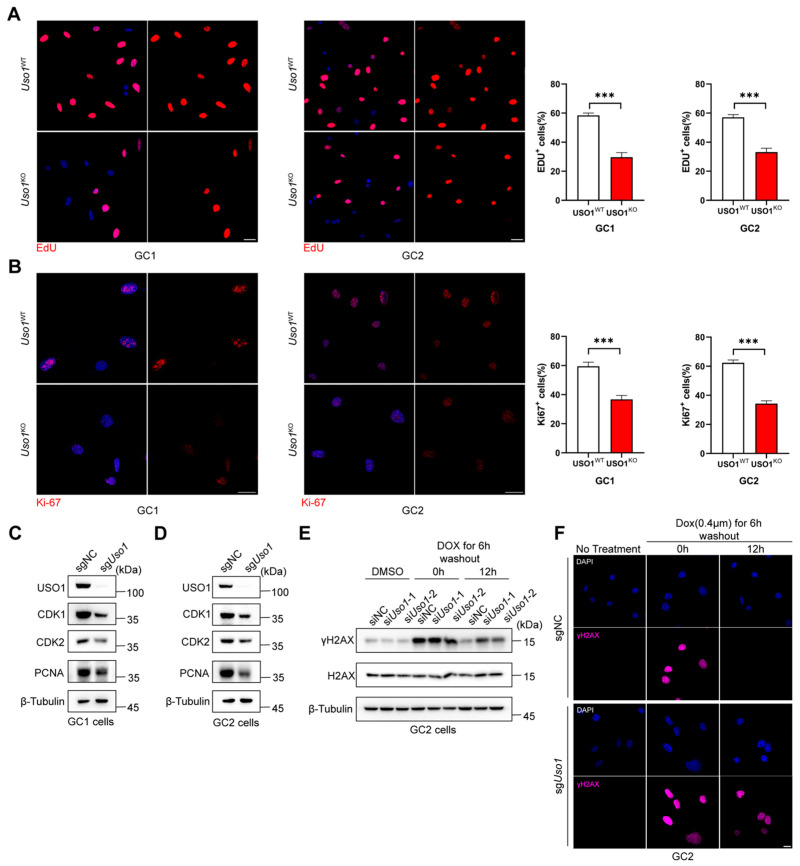
*Uso1* knockout represses cell proliferation and impairs DNA damage repair in male germ cells. (**A**) Proliferation ability was examined using an EdU incorporation assay in GC1 and GC2 cells. Scale bars = 25 μm. The quantitative results are presented in the right panel. Values are expressed as the mean  ±  SD from three independent experiments (two-tailed Student’s *t*-test, *** *p*  <  0.001). (**B**) Immunofluorescent staining of Ki-67 in GC1 and GC2 cells. Scale bars = 25 μm. The quantitative results are presented in the right panel. Values are expressed as the mean  ±  SD from three independent experiments (two-tailed Student’s *t*-test, *** *p*  <  0.001). (**C**,**D**) The expression of the cell-cycle-associated proteins CDK1 and CDK2, as well as the proliferation-related protein PCNA, were analyzed using Western blotting for GC1 and GC2 cells. β-Tubulin was used as the internal standard. (**E**) Immunoblot analysis of γH2AX and H2AX in GC2 cells treated with dimethyl sulfoxide (DMSO) or doxorubicin. (**F**) Immunofluorescent staining of γH2AX in GC2 cells treated with DMSO or doxorubicin. Blue—DAPI, red—γH2AX. Scale bar = 15 μm.

To elucidate the effect of USO1 on testis development, we generated *Uso1*-knockout GC1-spg and GC2-spd cell lines. We observed that *Uso1*^KO^ cells grew considerably slower than WT cells. An examination of Ki-67 by immunofluorescence staining showed lower levels in *Uso*1^KO^ cells than in *Uso1*^WT^ cells (Figure 2A), and the percentage of EDU-positive cells was significantly reduced (Figure 2B). Consistently, PCNA activity decreased in *Uso1*^KO^ cells compared with *Uso1*^WT^ cells (Figure 2C,D). These data suggest that USO1 deficiency impairs the proliferation ability of germ cells.

During meiosis, from the leptotene stage to the zygotene stage, γH2AX accumulates in the nucleus with the generation of DNA double-strand breaks (DSBs). As the DNA repair process proceeds, γH2AX on the autosomes completely disappears at the pachytene stage. Doxorubicin (DOX), one of the most widely used anticancer agents, induces DNA double-strand breaks. To evaluate if the *Uso1* knockout impaired meiosis progression, we treated the mouse spermatocyte line GC2 with DOX for 6 h. Immunoblot analyses and immunofluorescence assays were performed using anti-γH2AX at 0 h and 12 h after drug washout, respectively (Figure 2E,F). These showed that the depletion of USO1 did not affect the response of γH2AX at 0 h. However, γH2AX could not recover to the same level as that of the control group 12 h after the drug elution. These data indicated that USO1 knockdown resulted in abnormal spermatocyte DNA damage repair and could further lead to meiotic cell cycle arrest. The immunoblotting results showed that after the *Uso1* knockout, the expression levels of CDK1 and CDK2 decreased, indicating that USO1 deficiency has an impact on the germ cell cycle.

### 2.3. Uso1 Knockout Induces Cell Cycle Arrest and Apoptosis in GC1 and GC2 Cells

**Figure 3:**
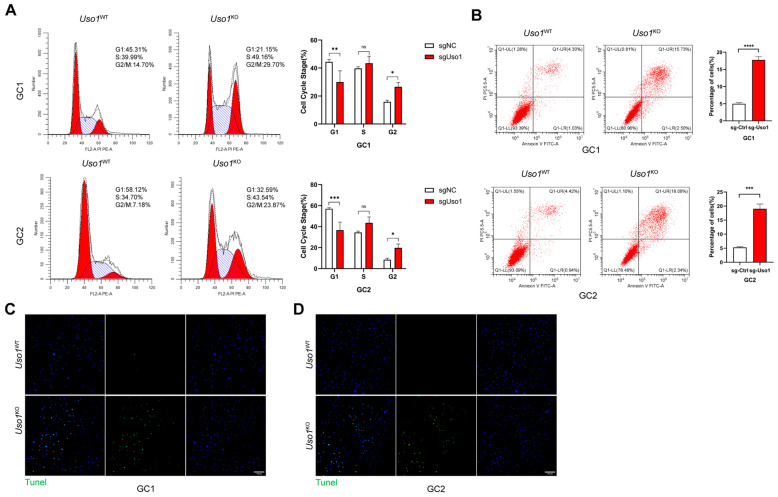
USO1 affects cell cycle distribution and apoptosis of GC1 and GC2 Cells. (**A**) Cell cycle analysis of sgNC and sg*Uso1* in GC1 and GC2 cells. (**B**) Apoptosis assays, with sgNC and sg*Uso1* in GC1 and GC2 cells. (**C**,**D**) Representative images of TUNEL staining (20×) obtained from sgNC and sg*Uso1* in GC1 and GC2 cells. Scale bar = 20 μm. All experiments were independently replicated thrice. Statistical significance was determined as follows: * *p* < 0.05, ** *p* < 0.01, *** *p* < 0.001, **** *p* < 0.0001 and ns: non-significant.

To investigate the impact of USO1 deficiency on cell cycle regulation, a quantitative flow cytometric analysis was conducted. The experimental data revealed that USO1 deficiency induced substantial cell cycle progression arrest at the G2/M checkpoint in both GC1 and GC2 cell lines. Specifically, a flow cytometric quantification demonstrated a significant increase in G2/M phase-detained cells in the *Uso1*-knockout GC1 populations (29.70%) compared with wild-type controls (14.70%). This cell cycle perturbation pattern was consistently observed in GC2 cells, with *Uso1*^KO^ cell populations showing comparable G2/M phase arrest (Figure 3A). The concordant results from both cellular models substantiated the critical regulatory role of USO1 in mitotic phase transition.

To investigate the potential pro-apoptotic effects of USO1 deficiency, Annexin V-FITC/PI dual-labeling flow cytometry was employed for a quantitative assessment of apoptotic progression. The analysis revealed that the *Uso1* knockout significantly increased the percentage of apoptotic cells. In *Uso1*^KO^ GC1 cells, the apoptosis rate at early and late stages was 2.50% and 15.73%, respectively, in contrast to 5.33% total apoptotic cells observed in *Uso1*^WT^ cells. This apoptotic potentiation was corroborated in GC2 cells, where *Uso1*^KO^ induced a 2.34% early apoptosis rate and 18.08% late apoptosis rate compared with 5.36% total apoptotic cells in *Uso1*^WT^ controls (Figure 3B). Additionally, TUNEL assays demonstrated significantly higher apoptosis indices in both *Uso1*^KO^ GC1 and GC2 cell lines relative to wild-type populations (Figure 3C,D).

### 2.4. Knockdown of Uso1 Causes Transcriptional Dysregulation in GC1 and GC2 Cells

**Figure 4:**
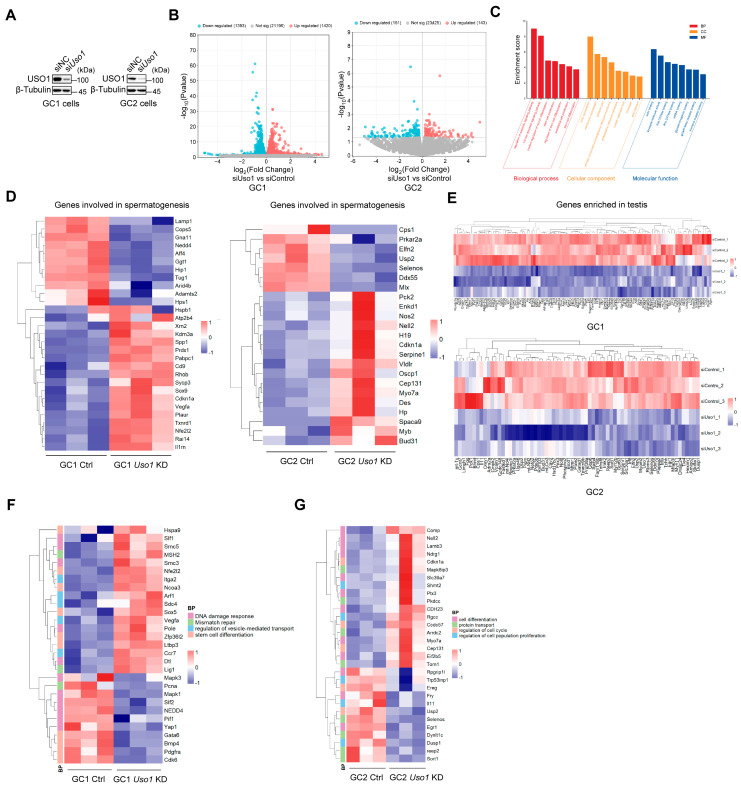
Knockdown of *Uso1* causes transcriptional dysregulation. (**A**) Western blotting showing the downregulation of *Uso1* in si*Uso1*-transfected GC1 and GC2 cells compared with their respective controls, with β-tubulin serving as a loading control. (**B**) Volcano plots depicting the quantity of significantly differentially expressed genes (DEGs) in GC1 and GC2 cells. Upregulated genes are indicated by red dots, while downregulated genes are marked by blue dots. Genes that are not DEGs are presented as dark-gray dots. The P threshold (=0.05) is indicated by gray horizontal dashed lines. (**C**) GO enrichment analyses of biological processes (BPs), cellular components (CCs), and molecular functions (MFs) were performed on 2813 DEGs of GC1 cells between the siControl and si*Uso1* groups. (**D**,**E**) Heatmap of genes associated with spermatogenesis differentially expressed between the siControl and si*Uso1* groups by RNA sequencing of GC1 and GC2 cells. The number of genes enriched in the testes decreased in GC1 and GC2 *Uso1*-knockdown groups. (**F**,**G**) Hierarchical clustering analysis of four functionally annotated gene clusters revealing divergent expression profiles between siControl and si*Uso1* groups in GC1/GC2 cells. The color gradient, ranging from red to blue, represents relative gene expression levels from high to low.

To acquire a deeper understanding of the molecular functionality of USO1 in spermatogenesis, genome-wide RNA sequencing was performed to systematically identify transcriptional alterations in *Uso1*-knockdown cells (Figure 4A). The RNA sequencing of *Uso1*-knockdown germ cells identified 2813 differentially expressed genes (DEGs) in GC1 cells, and 1393 genes were downregulated and 1420 genes were upregulated. Similarly, *Uso1* depletion in GC2 cells led to the downregulation of 151 genes and the upregulation of 143 genes (Figure 4B). A GO analysis of the 2813 differentially expressed genes (DEGs) in GC1 cells revealed that clustering occurred in the apoptotic signaling pathway during cell proliferation and differentiation, which was consistent with previously observed phenotypes (Figure 4C).

The expression of genes involved in spermatogenesis, including spermatogonial stem cell maintenance [21,22], sperm morphology [23], and fertilization [24], was dramatically changed in *Uso1*-knockdown cells (Figure 4D). The expression of genes exclusively or predominantly expressed in the testes was substantially diminished in GC1 and GC2 *Uso1*-knockdown cells (Figure 4E). Furthermore, the expression levels of certain key genes involved in the DNA damage response, mismatch repair, and stem cell differentiation dramatically changed in GC1 cells in the absence of *Uso1*, and genes related to the known function of USO1 in vesicle-mediated transport also underwent significant changes (Figure 4F). In *Uso1*-knockdown GC2 cells, genes involved in protein transport, cell differentiation, regulation of the cell cycle, and proliferation were also considerably changed (Figure 4G).

As evidenced by these findings, *Uso1* knockdown in germ cells induces transcriptional perturbation, particularly in crucial meiotic genes, potentially resulting in compromised germ cell proliferation.

### 2.5. USO1 Associates with Centriolar Satellite Proteins

**Figure 5:**
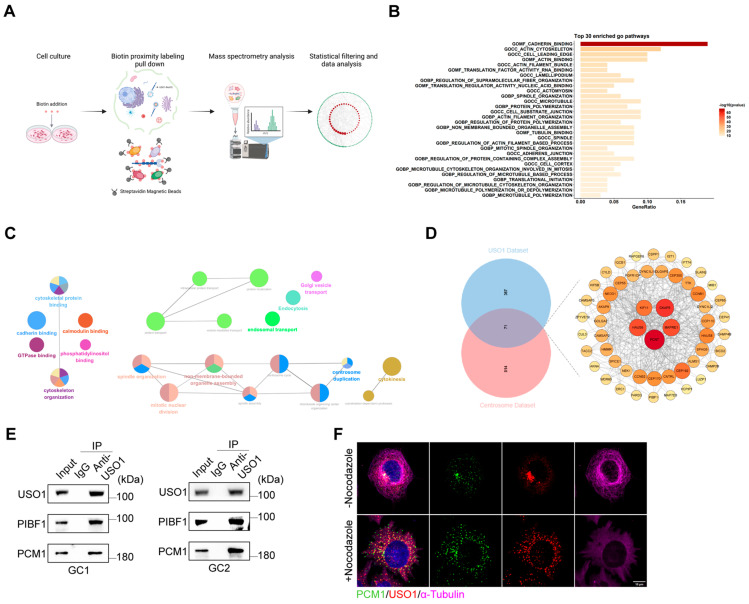
The interacting proteins of USO1 are enriched in biological processes related to centriolar satellites. (**A**) Schematic representation of cell- and subcompartment-specific BioID2 proximity-dependent biotinylation in 293T cells. (**B**) GO pathway analysis of proteins interacting with USO1. (**C**) Network diagram of enrichment results using Cytoscape software with integrated ClueGO functions. (**D**) Venn diagram of USO1 and centrosome datasets. The intersection of the two datasets is presented. (**E**) Co-immunoprecipitation (CoIP) of GC1 and GC2 cell lysates. The proteins were incubated with anti-PIBF1, an anti-PCM1 antibody, or rabbit IgG. Endogenous USO1 co-immunoprecipitated with PIBF1 and PCM1 in cell lysates. (**F**) Stable HeLa cells were treated with nocodazole and stained as indicated. Scale bar = 10 μm.

To gain a deeper insight into the molecular mechanisms of USO1 and explore its biological function in male reproduction, we performed proximity labelling proteomics for USO1 using 293T cells (Figure 5A). USO1 was conjugated to biotin ligase, which biotinylates proteins based on their proximity. The constructs were transiently transfected into HEK293T cells. Proteins labeled with biotin were subjected to streptavidin pulldown, followed by mass spectrometry.

We identified 438 interactors for USO1, with a fold change of >2.0. To identify the over-represented biological processes and molecular functions in the interactome list, we performed GO enrichment analyses. The most enriched pathways mainly focused on the biological processes related to the cytoskeleton, including cadherin binding, the actin cytoskeleton, and the cell leading edge (Figure 5B). Concurrently, we used ClueGO, a Cytoscape (v3.10.2) plug-in, to decipher functionally grouped gene networks, and found that a significant portion of the interacting proteins were enriched in centrosome-related biological processes, including spindle organization, centrosome duplication, and cytokinesis (Figure 5C). Previous studies have constructed a dataset of centrosomal and ciliary proteins using 58 bait proteins for proximity labeling combined with mass spectrometry [25]. A comparison revealed that USO1′s interacting proteins showed an extensive overlap with the centrosomal protein dataset (Figure 5D). We focused on two centriolar satellite proteins, PCM1 and PIBF1. Centriolar satellites (CSs) are membrane-free particles that aggregate around the centrosome and are dispersed in the cytoplasm. They are involved in a variety of cellular activities, such as microtubule organization and spindle maintenance, DNA damage repair, aggregate formation, and disaggregation. PCM1 was the first centriolar satellite protein to be identified and it accumulates in the centrosome through PIBF1, along with other centriolar satellite proteins. A co-immunoprecipitation (CoIP) analysis revealed the interaction between USO1 and both PCM1 and PIBF1. We also confirmed the co-localization of USO1 and PCM1 using immunofluorescence. Previous studies have shown that after microtubule depolymerization induced by treatment with the microtubule depolymerizing agent nocodazole or ice treatment, PCM1 can still interact with multiple satellite components and co-localize to form aggregates in the cytoplasm, indicating that the assembly of the satellite structure is independent of the microtubule network [26]. We found that the co-localization of USO1 and PCM1 also did not depend on the microtubule network, suggesting that USO1, as a vesicle-tethering protein, may play an important role in the assembly of satellite structures independent of the microtubule. Taken together, these data revealed that USO1 could be a potential component of centrosome satellite modulators.

## 3. Discussion

USO1 is a general vesicular transport factor responsible for the material transportation of the endoplasmic reticulum–Golgi route and for intercisternal transport in the Golgi stack. It is necessary for adjacent membrane fusion or the binding of the vesicles to the target membrane. It may act as a vesicular anchor by interacting with the target membrane and maintaining vesicular and target membranes in proximity. As one of the most extensively studied golgin proteins to date, its biological functions are implicated in numerous aspects of life processes. These include cell mitosis [14], autophagy [27], apoptosis [28,29,30], and directional migration [31,32] as well as intracellular lipid and protein transport [33,34].

Previous studies have shown that USO1 is implicated in various tumor types, including hematological malignancies [35,36,37,38], colorectal and gastric cancer [39], lung adenocarcinomas (LUADs) [40], and liver cancer [41]. Studies on the function of USO1 in the development of these cancers have shown that USO1 plays an oncogenic role by promoting cell proliferation, evading apoptosis, and disrupting the cell cycle. The deregulation of USO1 decreased colon cancer cells in the G2-M phase [39]. However, the potential role and molecular mechanisms of USO1 in spermatogenesis and male fertility have not been investigated. In this study, we first detected the expression of USO1 in different tissues and found that USO1 was highly expressed in mouse testes. Our results indicate that USO1 is expressed during the entire murine spermatogenic process and its expression levels are high in spermatogonia and spermatocytes. This finding suggests that USO1 might play an important role in mammalian germ cells during spermatogenesis. As expected, a critical role for USO1 in spermatogonia and spermatocytes was observed when USO1 was deleted in these cells. Our functional experiments confirmed that USO1 plays a vital role in the regulation of germ cell proliferation, and the knockdown of *Uso1* disrupted the DNA damage repair process in GC2 cells. The knockdown of *Uso1* markedly delayed cell cycle progression. Our experiment demonstrated for the first time that USO1 is of great significance to the proliferative capacity of germ cells. This prompted us to explore the molecular mechanisms underlying these phenomena further. A transcriptome analysis disclosed that *Uso1* knockdown induced the dysregulation of numerous transcripts of testis-enriched and reproduction-related genes. We also identified many key genes that regulate the proliferation, differentiation, and meiosis of germ cells. In summary, USO1 could be a regulator of normal reproductive function by modulating essential molecules associated with spermatogenesis.

Centriolar satellites contain numerous proteins that are responsible for centrosome maintenance [42], ciliogenesis [43], and some other functions that are yet to be discovered. Previous studies have shown that the interacting proteins CEP131 and CCDC13 of PCM1 are important CS molecules that are involved in chromosome segregation [44,45]. Similar to our results, DNA damage and a suppressed proliferation rate were observed in cells in which CEP131 or CCDC13 were knocked out, with increased γH2AX foci. USO1 has been proven to maintain mitotic spindle function because its depletion leads to the formation of multipolar spindles and incorrect chromosome segregation, ultimately resulting in failed cytokinesis [14]. Cells depleted of CEP131 or CCDC13 display similar phenotypes to USO1, including spindle collapse and micronuclei formation [44,45]. In our research, there was a strong correlation between USO1 and centriolar satellite proteins, and the interaction and co-localization of USO1 with centriolar satellite proteins were confirmed. However, there is a lack of direct evidence to elucidate the mechanism by which USO1 regulates CS, thereby causing cell cycle arrest, proliferation inhibition, and, ultimately, cell apoptosis. We hypothesized that the following mechanism may exist: USO1 has been reported to associate with spindle poles throughout mitosis, and its N-terminal armadillo domain mediates an association with the γ-tubulin-containing complex that provide sites for microtubule nucleation and anchoring, indicating that USO1, as a tethering protein, may be responsible for recruiting centriolar satellite components to the centrioles to exert their physiological functions. A functional deficiency of USO1 might affect the normal function of CS, thereby leading to the instability of the centrosome, which then blocks cell cycle progression and causes the eventual apoptosis of the cell.

In conclusion, our data indicated that USO1 is expressed in all types of spermatogenic cells and regulates the expression of key genes involved in spermatogenesis. Moreover, there was a novel observation for golgin proteins, which we first found in relation to CS components. USO1 may affect the cell cycle and cell growth by regulating CS proteins. Elucidating the potential mechanism by which USO1 plays a role in the male reproductive process enriches our understanding of the function of golgin proteins in male fertility.

## 4. Materials and Methods

### 4.1. Histological Analyses and Immunostaining

Testes from wild-type (WT) male mice were immediately dissected by cervical dislocation and immersion-fixed in 4% paraformaldehyde (Sigma-Aldrich, St. Louis, MO, USA at 4 °C for 24 h. Following gradient ethanol dehydration, tissues were paraffin-embedded and sectioned (5 μm) using a Leica microtome (Leica Biosystems, Nussloch, Germany). Deparaffinized sections underwent heat-mediated antigen retrieval in a 10 mM sodium citrate buffer (pH 6.0) at 95 °C for 15 min, followed by cooling to an ambient temperature. After permeabilization with 0.1% Triton X-100 in PBS (PBST), sections were blocked with 10% bovine serum albumin (BSA; 1 h, RT) and incubated with primary antibodies (4 °C, 16 h; see Supplementary Table S1). Following the PBST washes, sections were counterstained with fluorophore-conjugated secondary antibodies and 4′,6-diamidino-2-phenylindole (DAPI; 1 h, RT), then mounted for imaging using a Leica TCS SP8 confocal microscope (LAS X software, v3.7.4).

### 4.2. Immunofluorescence Staining

Cells were seeded at optimal densities and cultured overnight in a 37 °C humidified incubator with 5% CO_2_. Following fixation with 4% paraformaldehyde (PFA) for 15 min at room temperature, the samples were washed three times with PBS containing 0.1% Tween-20 (PBST). Permeabilization was performed using 0.1% Triton X-100 for 10 min, followed by blocking with 10% bovine serum albumin in PBST for 1 h at room temperature. Primary antibodies (detailed in Supplementary Table S1) were applied and incubated at 4 °C overnight. Specimens were then incubated with Alexa Fluor 488/594-conjugated secondary antibodies (Supplementary Table S1) and counterstained with DAPI (Solarbio, Beijing, China) for nuclear visualization for 60 min at room temperature. Imaging was conducted using a Leica TCS SP8 confocal laser scanning microscope (Leica Microsystems, Mannheim, Germany).

### 4.3. Western Blotting

Tissue and cellular proteins were extracted using a radioimmunoprecipitation assay (RIPA) buffer. Cellular lysates were mixed with a Laemmli buffer containing β-mercaptoethanol and denatured by heating at 95 °C for 10 min. Equal protein quantities (15–20 μg) were resolved using SDS–polyacrylamide gel electrophoresis (SDS-PAGE) and electrotransferred onto polyvinylidene difluoride (PVDF) membranes (Merck Millipore). The membranes were blocked with 10% skim milk in Tris-buffered saline containing 0.05% Tween-20 (TBST) for 2 h at an ambient temperature. Following overnight incubation with primary antibodies (Supplementary Table S1) at 4 °C, the membranes were washed thrice with TBST and probed with horseradish-peroxidase (HRP)-conjugated secondary antibodies (1:2000 dilution) for 1 h at room temperature. Protein bands were visualized using an enhanced chemiluminescence (ECL) substrate (Smart Life Sciences, Changzhou, China), with the exposure times optimized for signal intensity.

### 4.4. Establishment of Stable Uso1-Knockout Cells

Single-guide RNAs (sgRNAs; sequences in Supplementary Table S2) were cloned into the lentiCRISPRv2-dCas9 backbone (plasmid #112233; Addgene, Cambridge, MA, USA) using standard restriction enzyme-based cloning. Lentiviral particles were produced by co-transfecting HEK293T cells with the engineered lentiCRISPRv2 plasmids, psPAX2 packaging plasmid, and pMD2.G envelope plasmid (Genechem, Shanghai, China) at a 4:3:1 mass ratio. Viral supernatants were harvested 48–72 h post-transfection. The GC1 mouse spermatogonial cell line (SCSP-5254) and GC2 mouse spermatocyte cell line (SCSP-5055) were procured from the China Cell Bank/Stem Cell Bank. GC1 and GC2 cells were transduced with lentiviral particles in a polybrene-containing medium. Stable cell lines were selected using a 72 h puromycin treatment (2 μg/mL) with selection efficiency verified by parallel non-transduced controls.

### 4.5. Small Interfering RNA (siRNA)-Mediated Knockdown

Gene-specific siRNAs targeting *Uso1* (sequences detailed in Supplementary Table S2) and non-targeting control siRNAs were synthesized by Tsingke Biotechnology Co., Ltd. (Beijing, China). For transfection, GC1 and GC2 cells were seeded at 2 × 10^5^ cells/well in an antibiotic-free medium 24 h prior to reverse-transfection. siRNA-Lipofectamine 2000 complexes (Invitrogen, Carlsbad, CA, USA) were prepared at a 1:2.5 (pmol siRNA–μL reagent) ratio in an Opti-MEM reduced serum medium (Gibco, Thermo Fisher Scientific, Waltham, MA, USA), followed by a 20 min incubation at room temperature. Post-transfection incubation was maintained for 48 h at 37 °C with 5% CO_2_.

### 4.6. Cell Cycle Analysis and Cell Apoptosis Assay

Cells were harvested by centrifugation at 300× *g* for 5 min at 4 °C and washed thrice with ice-cold phosphate-buffered saline (PBS). For cell cycle profiling, pellets were fixed in 75% pre-chilled ethanol at −20 °C for 24 h, then processed using a Cell Cycle Detection Kit (MedChemExpress, Monmouth Junction, NJ, USA) following manufacturer’s protocol, including RNase A treatment and propidium iodide (PI) staining. An apoptosis analysis was performed using dual-staining with Annexin V–fluorescein isothiocyanate (FITC) and PI via an Apoptosis Detection Kit (Solarbio, Beijing, China). The data analysis was performed using FlowJo v10.8 software.

### 4.7. RNA Sequencing Analysis

Total RNA was isolated from GC1 and GC2 cells (control and *Uso1*-knockdown groups) using TRIzol Reagent (Invitrogen, Carlsbad, CA, USA). cDNA libraries were constructed, and RNA sequencing was conducted by Novogene Biotechnology Co., Ltd. (Beijing, China). The resulting RNA-seq data were analyzed to identify differentially expressed genes (DEGs). A GO function analysis histogram was plotted using https://www.bioinformatics.com.cn (accessed on 2 November 2024), an online platform for data analysis and visualization.

### 4.8. Proximity-Dependent Biotin Identification (BioID) Coupled to Affinity Capture and Mass Spectrometry (MS)

#### 4.8.1. Cloning and Constructs

Full-length human *Uso1* was amplified via PCR using the primers 5′→3′ forward GGCCTGTTAACCGGTATGAATTTCCTCCGCGGGGT and 5′→3′ reverse CCGAATTCGAATCCGGATATGATCTAGATCCTTGCCA, which were inserted into pcDNA3.1(-)-MCS-13xLinker-BioID2-HA(Addgene) via a restriction digest using Age1 and BsmBI-v2.

#### 4.8.2. Biotin Proximity Labeling Pulldown and Mass Spectrometry

Transiently transduced 293T cells were cultured in 10 cm dishes and incubated for 48 h in biotin-depleted media before biotin addition. Biotin (50 μM) was added to the cell culture media and incubated overnight. The subsequent pulldown protocol was drawn largely from Roux et al. [46].

## Data Availability

The RNA-seq raw data were uploaded to the NCBI database with the accession number PRJNA1227761. The datasets used and/or analyzed during the current study are available from the corresponding author upon reasonable request.

## Supplementary Materials

The following supporting information can be downloaded at: https://www.mdpi.com/article/10.3390/ijms26094274/s1.
